# Bilateral Endogenous Endophthalmitis Secondary to Staphylococcus aureus Bacteremia

**DOI:** 10.7759/cureus.72937

**Published:** 2024-11-03

**Authors:** Fernanda N Susanna, Julia C Bastian, Maria Fernanda A de Sá Carricondo, Luiza S Santos Parra

**Affiliations:** 1 Ophthalmology, Hospital das Clínicas da Faculdade de Medicina da Universidade de São Paulo, São Paulo, BRA

**Keywords:** bacterial endophthalmitis, bilateral endophthalmitis, catheter-related complications, infective endophthalmitis, mssa bacteremia

## Abstract

Bacterial endogenous endophthalmitis is caused by a breach of the blood-ocular barrier by pathogens originating from distant sites. It is a rare cause of endophthalmitis and can lead to devastating outcomes without prompt and adequate treatment. We report the case of a 50-year-old woman with a history of type II diabetes mellitus who experienced an episode of acute myocardial infarction complicated by an acute exacerbation of chronic kidney disease, catheter-related infection, bloodstream infection, bacterial endocarditis, and bilateral endogenous endophthalmitis confirmed by blood culture and bilateral vitreous culture showing growth of methicillin-sensitive *Staphylococcus aureus* (MSSA). Vision deteriorated to loss of light perception despite aggressive systemic antibiotic therapy and three bilateral intravitreal injections of vancomycin, ceftazidime, and dexamethasone. Due to the severity of her clinical status, vitrectomy was contraindicated. This report presents a rare case of multi-organ infection and damage involving both eyes, which required multidisciplinary follow-up and carefully coordinated management in a patient with a life-threatening condition.

## Introduction

Bacterial endogenous endophthalmitis (BEE) is caused by a breach of the blood-ocular barrier by pathogens originating from distant sites. It is relatively rare, accounting for only 2-8% of endophthalmitis cases [1], with 12% of these being bilateral [2], and it can lead to devastating outcomes without prompt and adequate treatment.

The pathophysiology of BEE typically involves ocular infection due to persistent bacteremia from septic conditions with distant foci such as the liver, lung, or endocarditis. This is caused by pathogenic agents with virulence factors for septic embolization, with *Staphylococcus aureus* being the primary causative organism responsible for up to 25% of cases [3]. However, other bacteria may also be involved in BEE, including group B *Streptococcus*,* Streptococcus* *pneumoniae*, *Klebsiella*, and *Escherichia *​​​​*coli*, making the isolation of the pathogen through blood culture and vitreous culture critically important.

Risk factors for the development of BEE include prolonged hospitalization, invasive devices, diabetes mellitus, immunosuppression, bloodstream infection, septic conditions with multiple foci, and a history of injectable drug use. However, patients with no known risk factors or who are immunocompetent may also develop the disease.

As BEE is a rare condition, no established guidelines exist for its treatment. Collaboration with a medical team is often essential, as it may present a significant mortality rate. We report a case of bilateral endogenous endophthalmitis due to methicillin-sensitive *Staphylococcus aureus* secondary to catheter-related bloodstream infection and endocarditis caused by the same agent. 

## Case presentation

A 50-year-old woman with a history of type II diabetes mellitus, systemic hypertension, and ischemic heart failure presented with an episode of acute myocardial infarction complicated by acute pulmonary edema, urinary tract infection, and exacerbation of chronic kidney disease requiring dialysis during hospitalization. Fifteen days after admission, the patient developed significant ocular pain, hyperemia, and decreased visual acuity in both eyes. After five days of treatment with prednisolone and atropine eye drops at the initial facility, she was referred to the ophthalmology department at Hospital das Clínicas of the University of São Paulo Medical School (HCFMUSP) for further evaluation and appropriate treatment.

**Figure 1:**
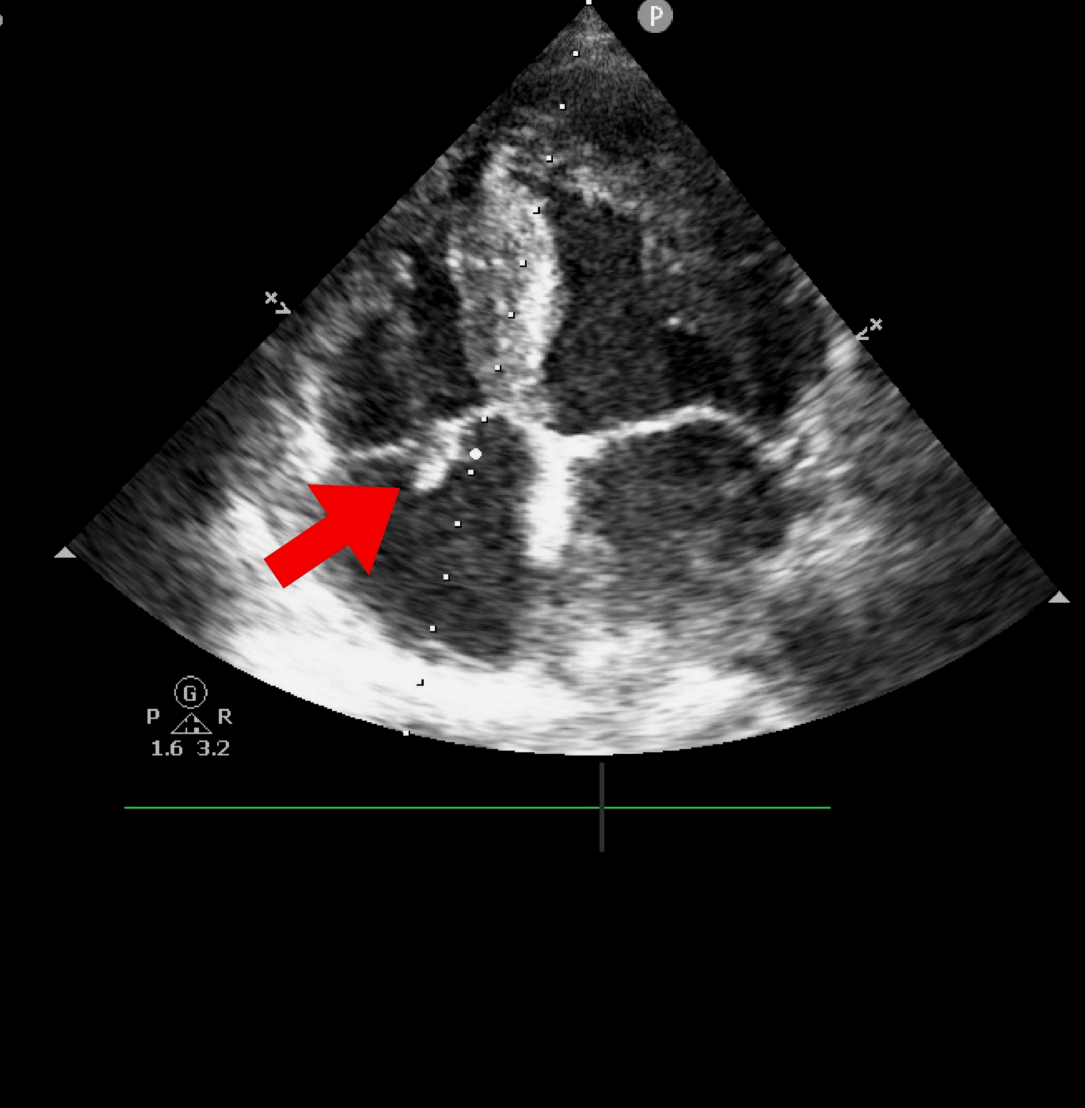
Transthoracic echocardiogram demonstrating the presence of vegetations on the tricuspid valve

**Figure 2:**
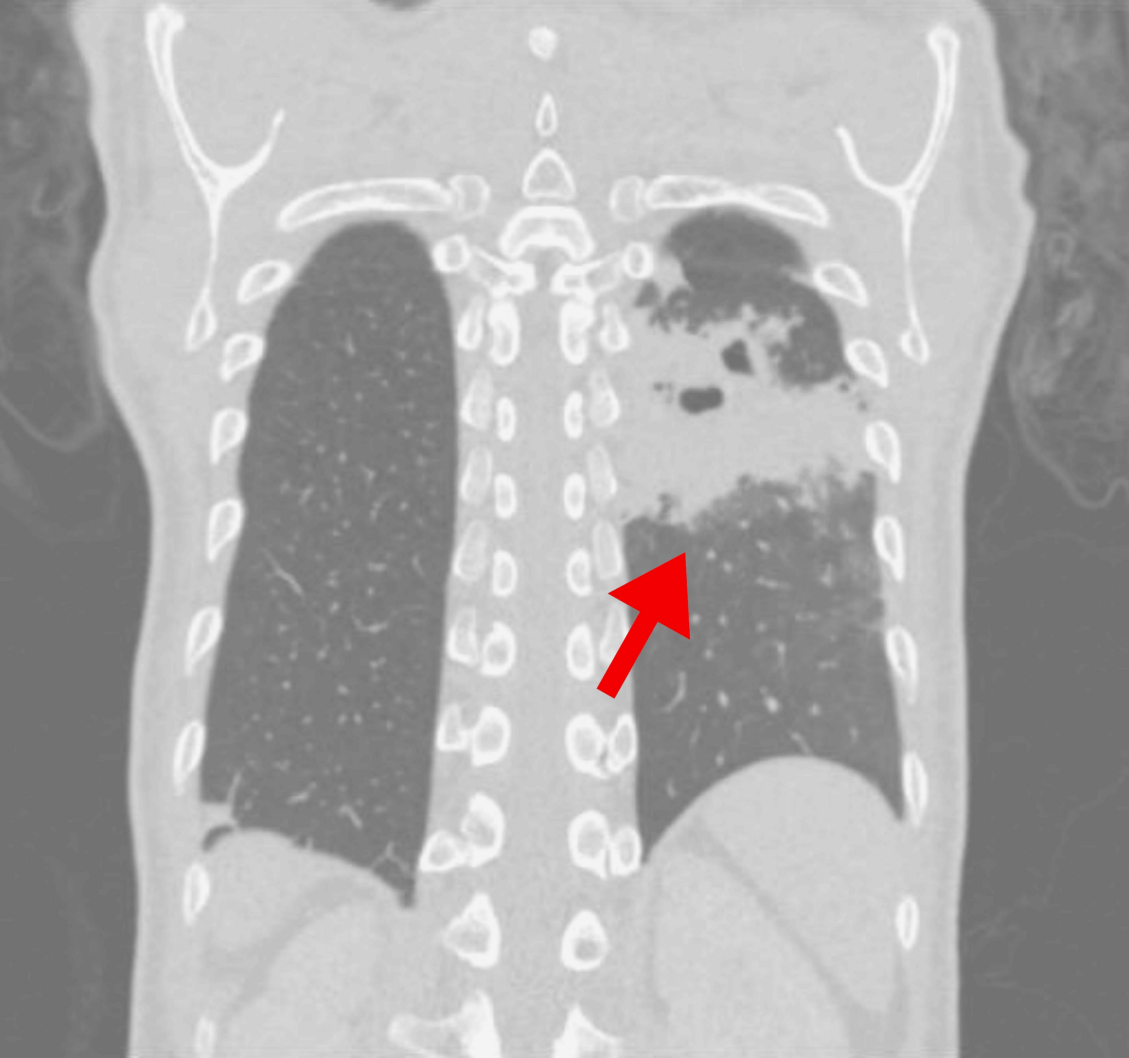
Chest tomography showing foci of septic embolization in the lungs, primarily in the left lung

Upon arrival, the patient was in a state of septicemia and was promptly taken to the intensive care unit, where she was diagnosed with catheter-related infection as well as bloodstream infection and bacterial endocarditis, confirmed by blood and catheter culture showing growth of methicillin-sensitive *Staphylococcus aureus* (MSSA) and the presence of vegetations on transthoracic echocardiogram (Figure 1). Additionally, she exhibited foci of septic embolization to the lungs on chest tomography upon admission (Figure 2).

During the initial ophthalmological evaluation, the patient exhibited bilaterally light perception acuity, miosis, moderate conjunctival hyperemia, corneal edema, increased intraocular pressure bilaterally, and hyphema in the left eye. Fundus examination was not possible due to pupillary seclusion and bilateral pupillary membrane. Ultrasound of the ocular bed revealed findings suggestive of endophthalmitis and bilateral lamellar retinal detachment. Given the characteristic clinical presentation in a patient with bloodstream infection, evidence of septic embolizations, and suggestive imaging, the primary hypothesis was bilateral endogenous endophthalmitis.

Due to strong clinical suspicion, an intravitreal puncture yielded methicillin-sensitive *Staphylococcus aureus* (MSSA) growth in culture, confirming the diagnosis. Concurrently, the patient received an intravitreal injection of vancomycin, ceftazidime, and dexamethasone. A second injection was performed 48 hours later due to a lack of response to treatment. She was also treated with hypotensive and mydriatic eye drops.

**Figure 3:**
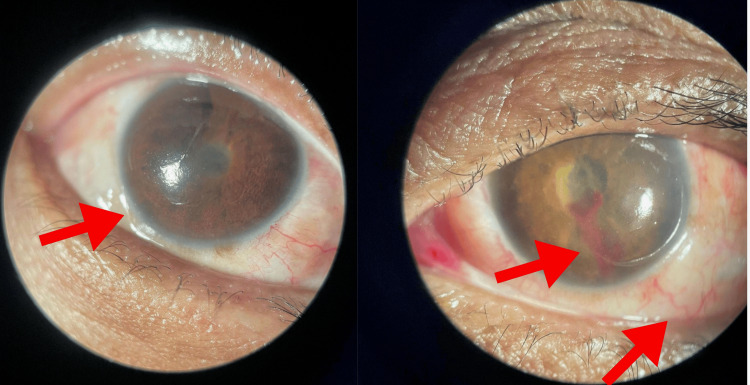
Slit lamp examination showed moderate conjunctival hyperemia and chemosis, corneal edema, pupillary seclusion, and pupillary membrane bilaterally, and hyphema on the left eye

After a week of local and systemic antimicrobial therapy, the patient experienced an improvement in hypopyon and bilateral pain; however, corneal edema, hyperemia, and low visual acuity persisted (Figure 3). Furthermore, a new intravitreal injection of antibiotics was contraindicated, and the patient was not in a clinical condition suitable for posterior vitrectomy (VVPP). The decision was made to continue with systemic antibiotic therapy using oxacillin, maintaining the combination with levofloxacin for better ocular penetration.

**Figure 4:**
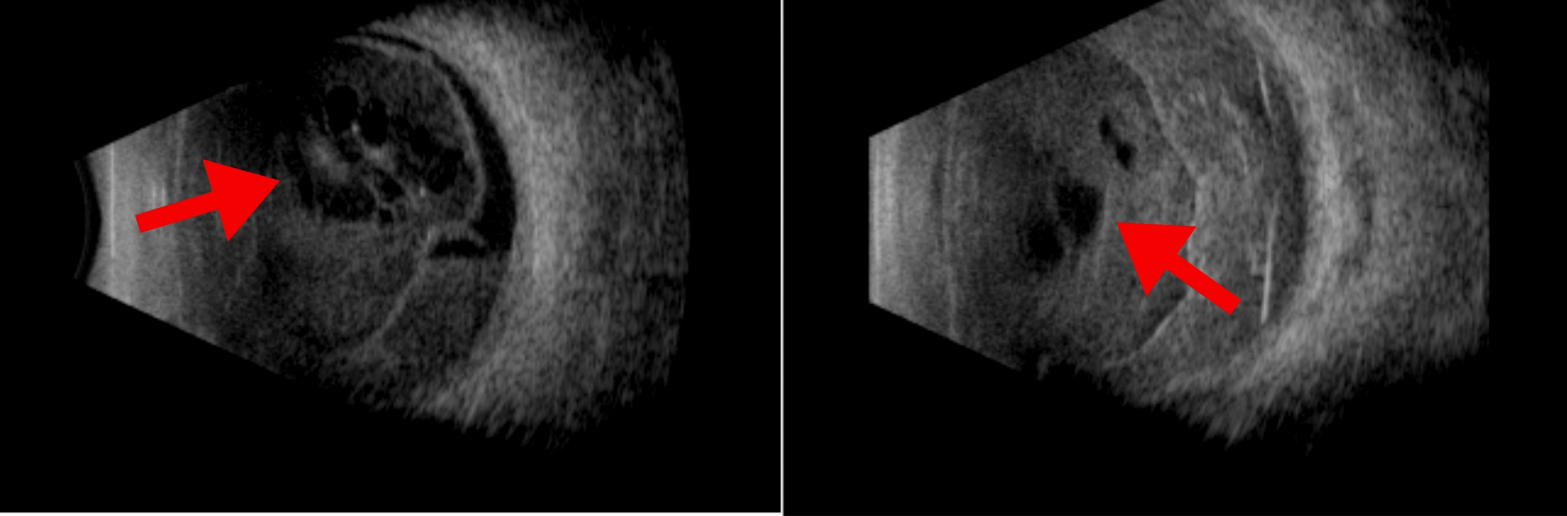
Ocular ultrasound showing intravitreal inflammatory/hemorrhagic process with vacuoles suggestive of endophthalmitis in both eyes

Thirty days after the last intravitreal injection, the patient had light perception acuity in the right eye and no perception in the left eye. Biomicroscopy revealed improvement in conjunctival hyperemia, while the other findings remained unchanged. A new ocular ultrasound showed an inflammatory/hemorrhagic process within the vitreous with vacuoles in both eyes (Figure 4). Given the possibility of ongoing infectious processes and visual acuity deterioration, a new intravitreal injection of antibiotics was administered.

The decision for conservative treatment was made due to the patient's clinical status, which included a new acute myocardial infarction during hospitalization, the need for continuous hemodialysis, persistent vegetations, and perforation of a cardiac valve. Cardiac surgery was also indicated; however, the patient was also not in suitable clinical condition. Due to the severity of her condition, the patient was jointly evaluated by the palliative care team, who confirmed the contraindication for extensive surgical procedures or other invasive interventions in addition to a do-not-resuscitate protocol. The patient was hospitalized for five months until clinical stabilization. She was eventually discharged and passed away at home two months later due to multiple organ dysfunction.

## Discussion

Our patient's ocular and systemic sequelae highlight the importance of early diagnosis of bacterial endogenous endophthalmitis as an indicator of potentially fatal systemic infections and the necessity for aggressive early treatment. This case involves a diabetic woman with MSSA bacteremia, septic emboli to the lungs, endocarditis with vegetations, multiple organ dysfunction, and bilateral BEE.

In a recent review of published cases of BEE over ten years, diabetes mellitus, along with malignancies, was identified as the most significant predisposing medical condition. The most prevalent extraocular infection foci were endocarditis, urinary tract infection, prolonged catheters, and infection related to dialysis access [4]. Our patient exhibited severe diabetes mellitus and multiple possible extraocular foci.

Endogenous endophthalmitis is a severe intraocular infection that can lead to significant morbidity and mortality. The mortality rates associated with this condition vary across studies, generally ranging from 3.7% to 21.1%, with some reports indicating rates as high as 52.2% [4]. One of the key risk factors for poor systemic outcomes is delayed diagnosis, as observed in our patient's case.

Previous retrospective studies have shown that misdiagnosis of BEE occurs in up to 50% of cases [5]. Classic symptoms such as ocular pain, conjunctival injection, and hypopyon in patients with risk factors for bloodstream infection should alert us to the possibility of endogenous endophthalmitis, even when fundus examination is not feasible. Other characteristics, such as eyelid edema, photophobia, and vitritis, may also be present [5,6]. 

Vision in BEE is preserved in less than half of cases, even with aggressive antibiotic therapy [7]. A retrospective study found that only 7% of patients without vitrectomy achieved a final visual acuity (VA) better than hand motion (HM) [8]. Therefore, unfortunately, our patients' outcomes align with the literature. Currently, there are no established guidelines for managing BEE; however, systemic therapy with third-generation cephalosporins or fluoroquinolones with good intraocular penetration is generally recommended [5]. In our case, levofloxacin was used. Most specialists also recommend intravitreal antibiotics, typically adhering to the classes and dosages used for postoperative endophthalmitis [4], including vancomycin, ceftazidime, and dexamethasone. This procedure can be repeated, as in our case. The outcomes of this treatment can positively influence visual prognosis if administered within the first 24 hours of symptom onset [9]. Unfortunately, despite her concerning signs and symptoms, the patient was only treated for ocular pain with corticosteroids and mydriatics at the initial facility. The five-day delay in diagnosing this patient may have contributed to the poor visual outcome observed at the end of treatment.

Pars plana vitrectomy is also a cornerstone of treatment [10]; however, it is rarely performed [4], likely because BEE is generally associated with severe systemic conditions that contraindicate surgical procedures. At no point during the treatment did our patient present a clinical condition suitable for this procedure, as her status progressively deteriorated until palliative care was indicated, with guidelines against invasive interventions. Despite not achieving visual recovery, aggressive management with systemic and intravitreal antibiotic therapy was essential for clinical stabilization and for halting the progression of visual loss.

## Conclusions

Endogenous bacterial endophthalmitis remains a significant and challenging condition. In severely ill patients, the threshold for suspecting endogenous endophthalmitis should be low. Classical symptoms, particularly hypopyon, should raise concern, as early recognition and prompt treatment are crucial to mitigating potential vision loss. Unlike most ocular infections, endogenous endophthalmitis can also pose a life-threatening risk, and multidisciplinary follow-up with the immediate initiation of systemic treatment is typically necessary. Future research should focus on refining diagnostic techniques and therapeutic strategies.
